# Analysis of the clinical characteristics of direct oral anticoagulants-associated atraumatic splenic rupture

**DOI:** 10.3389/fphar.2026.1777780

**Published:** 2026-03-06

**Authors:** Can Shi, Xia Wang, Siyi Zhang, Ren Guo, Tian Wu

**Affiliations:** Third Xiangya Hospital, Central South University, Changsha, China

**Keywords:** abdominal pain, atraumatic splenic rupture (ASR), direct oral anticoagulants (DOACs), pharmacovigilance, polypharmacy

## Abstract

**Objective:**

Atraumatic splenic rupture (ASR), though rare, is an adverse event linked to direct oral anticoagulants (DOACs). Given their widespread use and potentially fatal consequences if undiagnosed, heightened clinical awareness of DOAC-associated ASR is crucial. Our aim was to analyze the occurrence and clinical characteristics of ASR induced by DOACs.

**Methods:**

We conducted a retrospective analysis of all reported DOAC-associated ASR cases through 15 April 2025, without language restrictions.

**Results:**

A total of 27 patients (11 males and 16 females) were included with a median age of 64 years. Among them, apixaban (n = 17) was the most common DOAC, followed by rivaroxaban (n = 8) and dabigatran (n = 2), with atrial fibrillation (81.5%, n = 22) being the primary indication. The comorbidities observed among patients with DOAC-associated ASR risk included hypertension (25.9%), coronary heart disease (18.5%), malignancy (18.5%), and infections (18.5%). Among 27 patients, 11 (40.7%) received concomitant medications that may potentiate DOAC effects, with 5 patients taking four interacting drugs simultaneously. Only 4 of the 11 patients had documented anticoagulant dosages, half of which were full-dose regimens. Management included immediate DOAC cessation (100.0%), transfusion (77.8%), splenic artery embolization (44.4%), and splenectomy (70.4%) – with 31.6% of splenectomies representing salvage procedures following failed embolization. All patients were successfully discharged with no mortality.

**Conclusion:**

ASR is a potentially life-threatening but preventable DOAC complication. Early recognition—particularly in elderly patients with comorbidities and polypharmacy—and urgent imaging for abdominal pain are crucial for improving clinical outcomes.

## Introduction

Direct oral anticoagulants (DOACs), which act by specifically inhibiting thrombin or activated factor X (factor Xa) in the coagulation cascade, have become increasingly prescribed for thromboembolic prophylaxis and treatment due to their favorable pharmacokinetic profile and convenience (Long and Gottlieb, 2023; Hindley et al., 2023). Multiple large-scale randomized controlled trials (RCTs) have confirmed the satisfactory efficacy of DOACs along with their significant reduction in major bleeding risk (Patel et al., 2011; Agnelli et al., 2013; Granger et al., 2011; Connolly et al., 2009; Giugliano et al., 2013). However, it is noteworthy that despite an overall reduction in bleeding complications, atraumatic splenic rupture (ASR) is increasingly being recognized as a potential adverse effect associated with DOAC therapy (Ostos Perez et al., 2023).

ASR, which is defined as spontaneous capsular disruption of the spleen without significant trauma, is rare and life-threatening, especially if it is not immediately recognized (Renzulli et al., 2009). The mortality rate of ASR is approximately 12.2% (Renzulli et al., 2009). Recently, case reports have identified an association between DOAC use and ASR, a potentially catastrophic complication that remains poorly characterized in the literature.

Despite its rarity, DOAC-associated ASR warrants clinical vigilance due to widespread DOAC use and potentially fatal outcomes if undiagnosed (Writing Committee et al., 2024). In our study, we collected patients with ASR after treatments with DOACs and characterize the clinical features, risk factors, treatments, and outcomes, which will guide clinicians in early detection and appropriate management, ultimately reducing morbidity and mortality.

## Methods

### Search strategy

A comprehensive literature search was conducted in PubMed, Embase, Web of Science, the Cochrane Library, China National Knowledge Infrastructure (CNKI), VIP Database, and Wanfang Data from their inception through 15 April 2025, to identify studies on DOAC-associated ASR. The search strategy combined Medical Subject Headings (MeSH) terms and free-text words, including (“Apixaban” [MeSH] OR “Dabigatran” [MeSH] OR “Rivaroxaban” [MeSH] OR “Edoxaban” [MeSH] OR “Betrixaban” [MeSH] OR “DOACs” [All Fields] OR “NOACs” [All Fields] OR “Direct Oral Anticoagulants” [All Fields] OR “Novel Oral Anticoagulants” [All Fields]) AND (“Splenic rupture” [MeSH] OR “Spleen rupture” [All Fields]). There was no language restriction. Review articles, commentaries, animal studies, and duplicate reports were excluded.

### Data extraction

We used a self-designed table to extract the following patient characteristics: sex, age, nationality, anticoagulant regimen (including specific agent, dosage, and duration), indications for anticoagulation, underlying comorbidities, concomitant medications, risk factors for splenic rupture, clinical presentation, laboratory parameters (hemoglobin level, platelet counts, coagulation profile, and renal function), imaging findings, histopathological results (when available), treatments, and clinical outcomes.

### Diagnostic criteria for atraumatic splenic rupture

The diagnosis of ASR was confirmed through comprehensive evaluation incorporating clinical manifestations, imaging findings, and histopathological evidence when available. Key diagnostic components included: (1) acute onset of abdominal pain (typically left upper quadrant or diffuse) accompanied by signs of hemodynamic instability (systolic blood pressure <90 mmHg or a heart rate >100 bpm) and/or laboratory evidence of acute blood loss (hemoglobin decline ≥2 g/dL from baseline); (2) radiographic confirmation via contrast-enhanced abdominal CT demonstrating characteristic findings including splenic parenchymal disruption, subcapsular hematoma, or hemoperitoneum; (3) definitive exclusion of traumatic etiology through detailed history review and physical examination (Liu et al., 2019; Soeborg et al., 2024).

### Statistical analysis

Statistical analyses were performed with Statistical Package for Social Science (SPSS) version 27.0 (IBM Corp., Armonk, NY, United States). Categorical variables were expressed as percentages (%), while continuous variables were presented as medians with ranges. The causality of DOAC-associated ASR was assessed using the Naranjo Adverse Drug Reaction (ADR) Probability Scale.

## Results

### Patients’ information

**FIGURE 1:**
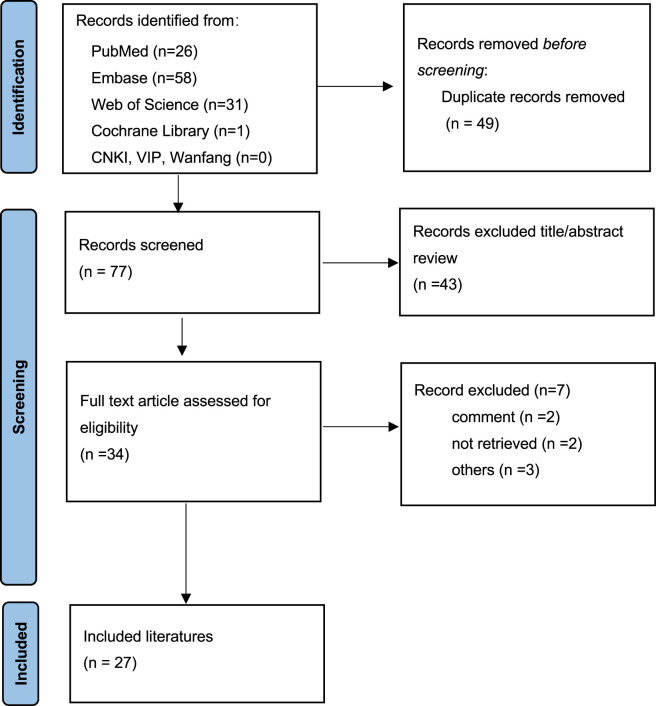
Flow chart of study selection process for reported cases of direct oral anticoagulants associated atraumatic splenic rupture.

A flow diagram for the study is provided in Figure 1. According to the inclusion and exclusion criteria, a total of 27 articles (Ostos Perez et al., 2023; Ahmed et al., 2020; Naseem et al., 2015; Bowers et al., 2024; Gonzva et al., 2014; High et al., 2024; Janke et al., 2020; Barnes et al., 2022; Kaufman and Ferraro, 2017; Kawazoe et al., 2022; Nagaraja et al., 2018; Widmer et al., 2021; Labaki and De Kock, 2022; Lopez Marcano et al., 2020; Lowry and Goldner, 2016; Moore et al., 2012; Mourato Nunes et al., 2018; Yau et al., 2020; Natarajan et al., 2021; Patnaik et al., 2021; Pietsch et al., 2021; Abdulrahman et al., 2021; Hetzler and Owens, 2021; Basnet et al., 2019; Carey and Nelatur, 2018; Amin et al., 2016; Abdelhady et al., 2018) involving 27 patients were included after full-text screening (Table 1). The basic information about these patients is summarized in Table 2. These patients (11 males and 16 females) with DOAC-associated ASR in our study were mainly from United States (48.1%) and Europe (37.0%), with a median age of 73 years (65–78 years). The DOACs were apixaban in 17 cases, rivaroxaban in 8 cases and dabigatran in 2 cases, 81.5% of which were prescribed for atrial fibrillation (AF) (22 patients). The most common comorbidity in the patients with DOAC-associated ASR was hypertension (25.9%), coronary heart disease (18.5%), malignant diseases (18.5%), and infectious diseases (18.5%). Among the 27 patients, 11 (40.7%) were prescribed concomitant medications that may potentiate the anticoagulant effect of DOACs. Specifically, 6 patients (22.2%) were on statins, 6 (22.2%) on aspirin and/or clopidogrel, 3 (11.1%) on amiodarone, and 1 (3.7%) on verapamil. Of the 11 patients receiving interacting medications, 3 used one such drug, 1 used two, 2 used three, and 5 used four concurrently. Among the 11 patients taking concomitant medications, only 4 had documented dosages of their anticoagulants. All 4 were on anticoagulation therapy for atrial fibrillation, with 2 of them (Naseem et al., 2015; Pietsch et al., 2021) receiving full-dose regimens.

**TABLE 1:** Summary of clinical information of 27 included patients.

References	Age/sex	Region	Drug	Indication	Dose	Duration	Symptoms	Risk factor	Concomitant medication	Treatment	Hospitalization day
Abdelhady et al. (2018)	62/f	Ireland	Apixaban	AF	—	6d	AP, Hypo	6d s/p PCI	Clopidogrel	Splenectomy and partial gastrectomy	18
Ahmed et al. (2020)	76/f	United States	Apixaban	AF	—	LT	AP, CP, nausea	Colonoscopy 16d prior	—	PRBCs, splenectomy	6
Abdulrahman et al. (2021)	77/f	—	Apixaban	AF	—	LT	AP, nausea, hypo	cancer^a^	—	PRBCs,PCC, splenectomy	10
Janke et al. (2020)	57/f	United States	Apixaban	PE	—	Recent	AP, hypo	Healthy	—	PRBCs, FFP, PCC, SAE, splenectomy	12
Amin et al. (2016)	68/f	United States	Rivaroxaban	DVT	—	—	AP, Hypo	Pancreatitis	—	PRBCs, SAE, splenectomy	21
López Marcano et al. (2020)	88/m	Spain	Apixaban	AF	—	LT	AP	HF, s/p GU surgery	Omeprazole, furosemide	PRBCs, splenectomy	11
Barnes et al. (2022)	58/f	United States	Apixaban	DVT	—	LT	AP, syncope, nausea, hypo	Cancer	—	PRBCs, PCC, SAE	—
High et al. (2024)	56/f	United States	Apixaban	AF	—	—	AP	RHD, IE	—	PRBCs, SAE	8
Yau et al. (2020)	66/m	AUS	Apixaban	AF	5 mg bid	LT	Syncope, AP, hypo	s/p Noro, EMCR	Amlodipine, telmisartan, aspirin, atorvastatin, metoprolol, citalopram	PRBCs, Cryo, FFP, platelets, PCC, SAE, splenectomy	6
Mourato Nunes et al. (2018)	78/m	Portugal	Apixaban	AF	—	—	Syncope, nausea, AP, CP, dyspnea, hypo	CLL, DM, HTN	—	PRBCs, FFP, PPC, Fbg, platelets, TXA, splenectomy	—
Gonzva et al. (2014)	67/m	France	Rivaroxaban	AF	15 mg qd	2 m	AP, hypo	Healthy	Betaxolol, rosuvastatin, flecainide, amlodipine	PCC, PRBCs, FFP, splenectomy	—
Lowry and Goldner (2016)	83/m	United States	Apixaban	AF	2.5 mg bid	LT	Dyspnea, hypo	CKD, COPD, HF,PH	Probenecid, finasteride, omeprazole	PRBCs, FFP, SAE, splenectomy	—
Widmer et al. (2021)	63/m	CHE	Apixaban	AF	—	LT	AP, syncope, hypo	CMML	Prednisone	SAE, splenectomy	13
Labaki and De Kock (2022)	64/m	Belgium	Rivaroxaban	AF	—	LT	Syncope, AP, dyspnea, hypo	HTN, Constip., AFL ablation	Amiodarone, bisoprolol, atorvastatin	PRBCs, FFP, platelets, splenectomy	—
Kaufman and Ferraro (2017)	65/m	United States	Apixaban	AF	—	LT	Syncope, AP dyspnea, hypo	AEP, ECV	Metoprolol, amiodarone	Splenectomy	—
Ostos Perez et al. (2023)	73/f	United States	Rivaroxaban	AF	15 mg qd	LT	AP, CP, hypo	HTN,HF	—	PRBCs, SAE	—
Hetzler and Owens (2021)	73/f	—	Rivaroxaban	AF	20 mg qd	LT	AP, CP	COPD	—	PRBCs, PCC, splenectomy	12
Patnaik et al. (2021)	82/f	United States	Apixaban	AF	2.5 mg bid	LT	AP	ESRD	Clopidogrel	Splenectomy	—
Natarajan et al. (2021)	81/m	United States	Apixaban	AF	—	LT	AP, hypo	HTN,DM, MG, PCI	Clopidogrel, aspirin	Conservative treatment	—
Carey and Nelatur (2018)	77/f	United Kingdom	Dabigatran	Takotsubo CM	—	1 day	AP	APE	—	Idarucizumab, PRBCs	—
Basnet et al. (2019)	86/m	United States	Apixaban	AF	5 mg bid	LT	AP, hypo	HTN,DM, CAD	Aspirin, atorvastatin, carvedilol, metformin	PRBCs, PCC, FFP, SAE, splenectomy	5
Kawazoe et al. (2022)	75/f	Japan	Apixaban	AF	—	LT	AP	—	—	SAE	—
Nagaraja et al. (2018)	77/f	AUS	Rivaroxaban	AF	20 mg qd	1 day	AP, hypo	Depression, ECV, HTN	Metoprolol, atorvastatin esomeprazole, quetiapine, amiodarone	PRBCs, Cryo, FFP, platelets, PCC,SAE	14
Bowers et al. (2024)	66/f	United States	Apixaban	AF	—	3 d	AP, hypo	3d s/p appendectomy, GCCA	—	PRBCs, FFP, splenectomy	30
Moore et al. (2012)	78/f	United States	Dabigatran	AF	100 mg bid	7d	AP, nausea	CAD, DM, PAD	—	PRBCs, FFP, SAE	—
Naseem et al. (2015)	66/m	Australia	Rivaroxaban	AF	20 mg qd	LT	AP, CP, dyspnea	CAD, pneumonia	Aspirin, atorvastatin, metoprolol, ramipril, digoxin	PRBCs, FFP, PCC, splenectomy	10
Pietsch et al. (2021)	76/f	Germany	Rivaroxaban	AF	20 mg qd	LT	AP	HTN	Verapamil	Splenectomy	—

_a_ suspected cancer; d, day; m, month; y, year.

AEP, acute eosinophilic pneumonia; AF, atrial fibrillation; AFL, atrial flutter; AP, abdominal pain; ASR, atraumatic splenic rupture; APE, acute pulmonary edema; AUS, Australia; CHE, Switzerland; CLL, chronic lymphocytic leukaemia; Constip. constipation; CP, chest pain; Cryo, Cryoprecipitated antihemophilic factor; DVT, deep vein thrombosis; ECV, electrical cardioversion; EMCR, extensive major arterial reconstruction; FFP, fresh frozen plasma; GCCA, appendiceal goblet cell adenocarcinoma; GU, gastric ulcer; HF, heart failure; HTN, hypertension; Hypo, hypotension; MG, myasthenia gravis; LT, long term; PAD, peripheral vascular disease; PE, pulmonary embolism; PH, pulmonary hypertension; PRT, portugal; PRBCs, packed red blood cells; RHD, rheumatic heart disease; SAE, splenic artery embolization; s/p, status post. Noro, Norovirus infection; TXA, tranexamic acid; United States, united states; VHD, valvular heart disease; VTE, venous thromboembolism.

**TABLE 2:** Characteristics of the 27 included patients with DOAC-associated ASR.

Parameter	Clinical features	Value
Sex	Male	11 (40.7)
	Female	16 (59.3)
Age	Years	73 (65–78)
Region	United States	13 (48.1)
	Australia	3 (11.1)
	Switzerland	1 (3.7)
	France	1 (3.7)
	Belgium	1 (3.7)
	United Kingdom	1 (3.7)
	Japan	1 (3.7)
	Portugal	1 (3.7)
	Spain	1 (3.7)
	Ireland	1 (3.7)
	Germany	1 (3.7)
	Unknown	2 (7.4)
DOACs	Apixaban	17 (63.0)
	Rivaroxaban	8 (29.6)
	Dabigatran	2 (7.4)
Indications	AF	22 (81.5)
	VTE	3 (11.1)
	Other	2 (7.4)
Accompanying diseases	Hypertension	7 (25.9)
	Coronary heart disease	5 (18.5)
	Malignant diseases	5 (18.5)
	Infections	5 (18.5)
	Diabetes	4 (14.8)
	Heart failure	3 (11.1)
	ESRD	1 (3.7)
Concomitant medications	Statins	6 (22.2)
	Aspirin/clopidogrel	6 (22.2)
	β-blockers	5 (18.5)
	Amiodarone	3 (11.1)
	Verapamil	1 (3.7)
Number of concomitant medications	1	3 (11.1)
	2	1 (3.7)
	3	2 (7.4)
	4	5 (18.5)

Data are presented as median (first quartile-third quartile) or number of subjects (%). AF, atrial fibrillation; ASR, atraumatic splenic rupture; DOACs, direct oral anticoagulants; VHD, valvular heart disease; VTE, venous thromboembolism.

### Clinical features

The clinical characteristics of the 27 patients with DOAC-associated ASR were summarized in Table 3. The time from anticoagulant initiation to symptom onset varied from 1 day to several years. Among these patients, 63% were on long-term anticoagulation (defined as ≥12 months of continuous use). All patients presented with abdominal pain, with some cases also exhibiting concurrent chest pain. Among the 27 patients, 18 developed hypotension, 6 experienced syncope, 5 reported nausea, and 5 manifested dyspnea. Identified risk factors for ASR were documented in 14 patients, including 5 with malignancies, 5 with infections, 3 following electrical cardioversion, and 1 after colonoscopy. ASR was preoperatively diagnosed in 9 patients, while 12 patients were diagnosed with ASR only during exploratory laparotomy. Additionally, 6 patients did not undergo surgery, or their surgical status was not described in the article. Based on the Naranjo ADR Probability Scale, the scores were distributed as follows: 13 patients (48.1%) scored 8 points (indicating a probable ADR), while 14 patients (51.9%) scored 5 points (suggesting a possible ADR).

**TABLE 3:** Clinical features of DOAC-associated ASR.

Parameter	Clinical features	Value
Time to ASR from taking DOACs	1∼7 days	6 (22.2)
	2 months	1 (3.7)
	Long term	17 (63.0)
	Unknown	3 (11.1)
Presenting symptoms	Abdominal pain	27 (100.0)
	Hypotension	18 (66.7)
	Syncope	6 (22.2)
	Nausea	5 (18.5)
	Dyspnea	5 (18.5)
	Chest pain	4 (14.8)
Examination	Abdominal CT	25 (92.6)
	Ultrasound	3 (11.1)
	Unknown	1 (3.7)
Risk factors for ASR	Malignant diseases	5 (18.5)
	Infections	5 (18.5)
	Electrical cardioversion/AFL ablation	3 (11.1)
	Colonoscopy	1 (3.7)
Pre/postoperative diagnosis	Preoperative	9 (33.3)
	Postoperative	12 (44.4)
	Unknown or not applicable	6 (22.2)
Laboratory indicators	Hemoglobin	​
	>10 g/dL	4 (14.8)
	7∼10 g/dL	7 (25.9)
	<7 g/dL	7 (25.9)
	Unknown	9 (33.3)
	INR	​
	<1.5	4 (14.8)
	1.5∼2.0	7 (25.9)
	Unknown	16 (59.3)
	APTT	​
	>35 s	2 (11.1)
	Normal	5 (18.5)
	Unknown	20 (74.1)

Data are presented as number of subjects (%). APTT, activated partial thromboplastin time; ASR, atraumatic splenic rupture; CT, computed tomography; DOACs, direct oral anticoagulants; INR, international normalized ratio.

### Laboratory tests

Of the 18 patients measured, 14 (51.8%) had hemoglobin levels below 10 g/dL, including 7 cases (38.9%) with severe anemia (<7 g/dL). Coagulation parameters revealed that International Normalized Ratio (INR) values between 1.5 and 2.0 in 7 patients (38.9%) – none exceeding the therapeutic threshold of 2.0 – while only 2 patients (11.1%) showed prolonged activated partial thromboplastin time (APTT >35 s). Details are shown in Table 3.

### Treatments and outcomes

The treatment and prognosis of the 27 included patients are summarized in Table 4. All patients immediately stopped DOACs. Among them, 12 patients (44.4%) underwent splenic artery embolization, and 19 patients (70.4%) required splenectomy, including 6 cases (31.6% of splenectomies) where splenic artery embolization failed, necessitating subsequent splenectomy. Transfusion support was provided to 21 patients (77.8%), consisting of packed red blood cells (n = 21), fresh frozen plasma (n = 11), prothrombin complex concentrate (n = 9), platelets (n = 4), and cryoprecipitate (n = 2). Three patients resumed anticoagulation without experiencing recurrent bleeding. All patients had favorable outcomes with no reported mortality cases.

**TABLE 4:** Treatments and outcomes of DOAC-associated ASR.

Parameter	Subgroup/Variable	Value
Treatments	Stop DOAC	27 (100.0)
	Transfusion therapy	21 (77.8)
	PRBCs	21 (77.8)
	FFP	11 (40.7)
	PCC	9 (33.3)
	Platelets	4 (14.8)
	Cryoprecipitate	2 (7.4)
	Splenic artery embolization	12 (44.4)
	Splenectomy	19 (70.4)
	Embolization of the splenic artery + splenectomy	6 (22.2)
Prognosis	Recovered and discharged	25 (92.6)
	Unknown	2 (7.4)
Resumption of anticoagulation	Yes	3 (11.1)
	No or unknown	24 (88.8)
Hospitalization day	1∼7 day	3 (11.1)
	8∼14 day	8 (29.6)
	>14 day	3 (11.1)
	Unknown	13 (48.1)

Data are presented as number of subjects (%). ASR, atraumatic splenic rupture; DOACs, direct oral anticoagulants; FFP, fresh frozen plasma; PCC, prothrombin complex concentrate; PRBCs, red blood cells.

## Discussion

ASR has recently been recognized as a bleeding complication of DOACs that is frequently overlooked yet potentially fatal in clinical practice. Our study identified Factor Xa inhibitors as the primary trigger of DOAC-associated ASR, with apixaban being the most frequently implicated. The latency period from anticoagulant initiation to ASR symptom onset varied widely, ranging from 1 day to several years, though most cases occurred during long-term anticoagulation therapy for AF. Notably, DOAC-associated ASR was more commonly reported in elderly patients (>73 years) and those of European/North American descent. The most prevalent comorbidities among DOAC-associated ASR patients included hypertension, coronary heart disease, malignancies, and infectious diseases. Of the 27 patients analyzed, 11 (40.7%) were taking concomitant medications that may potentiate DOAC effects. All patients presented with abdominal pain, and 92.8% required embolization or splenectomy; all patients achieved favorable outcomes following intervention.

The reported incidence of ASR is approximately 3.2% (Liu et al., 2019), with a concerning mortality rate reaching 12.2% (Renzulli et al., 2009). The clinical significance of ASR lies in the potential for delayed diagnosis to lead to persistent internal hemorrhage, hemodynamic instability, and ultimately fatal outcomes. Current research suggests that anticoagulants may increase splenic rupture risk by interfering with the spleen’s hemostatic mechanisms, inducing microtrauma and reactive macrophage infiltration within splenic tissue. This association is supported by systematic reviews, including Aubrey-Bassler and Sowers’ analysis of 613 cases identifying 47 drug-related ASR episodes (7.6%), including 21 anticoagulant-associated cases (Aubrey-Bassler and Sowers, 2012), and Renzulli et al.'s larger series of 845 ASR patients documenting 67 drug-induced instances, 22 of which involved anticoagulants (Renzulli et al., 2009). Apixaban, a direct factor Xa inhibitor, was demonstrated non-inferior to warfarin for stroke prevention in patients with atrial fibrillation as well as for management of venous thromboembolism, with a decreased risk of major bleeding, in the ARISTOTLE (Granger et al., 2011) and AMPLIFY (Agnelli et al., 2013) trials, respectively. Despite the reduced bleeding risk, our study suggested that Factor Xa inhibitors may be a primary trigger of ASR-related cases, with apixaban being the most frequently implicated. This may be related to apixaban having the highest hepatic metabolism proportion (75%) among DOACs (Chan et al., 2020), and the precise mechanisms require further investigation.

The spleen, as the body’s most vascularized lymphoid organ, becomes particularly vulnerable when pre-existing pathological conditions—such as infectious splenomegaly, vascular malformations, or infiltrative disorders—compromise its structural integrity (Akoury and Whetstone, 2025). This vulnerability is exacerbated by anticoagulation therapy through directly impairing hemostasis while (2) potentially accelerating subcapsular hematoma progression to frank rupture. In our study, 14 out of 27 patients (51.9%) had comorbid conditions that could lead to pathological splenic changes, such as malignancies or infections, while 11 patients (40.7%) were taking concomitant medications that may potentiate anticoagulant effects.

Until now, no guidelines for ASR treatment are available, and suggestions are derived from case reports. Splenectomy, splenic artery embolization, and transfusion therapy are the mainstays, which could be inferred from the above-mentioned cases. In our study, among the 12 patients who attempted embolization, 6 ultimately required splenectomy due to embolization failure, representing a 50% failure rate. The underlying mechanisms may involve two key factors: ① impaired tissue repair capacity in pathologically altered spleens, and ② the advanced age of the patient population (mean age 73 years). These findings imply that open splenectomy remains the definitive therapeutic option for anticoagulant-associated ASR, particularly in hemodynamically unstable patients.

This study has several important limitations. First, the case series component included only 27 patients, allowing only descriptive summaries without hypothesis testing, risk estimation, or adjustment for confounders. Second, the absence of a control group precludes distinguishing drug-related risks from those attributable to underlying conditions, concurrent illnesses, recent procedures, or coincidental associations—even in instances where Naranjo scores indicate a possible or probable relationship. Third, heterogeneity in case definitions, reporting completeness, and management strategies limits meaningful subgroup comparisons. Consequently, these findings should be interpreted as exploratory and serve primarily to inform the design of future large-scale, prospective studies.

## Conclusion

ASR may represent a critical yet potentially preventable complication of DOAC therapy. Enhancing clinical vigilance, especially in elderly patients with comorbidities and polypharmacy, coupled with prompt imaging for abdominal pain, could help mitigate adverse outcomes. Future research should focus on identifying biomarkers for early detection and exploring spleen-sparing management strategies through prospective studies.

## Data Availability

The original contributions presented in the study are included in the article/supplementary material, further inquiries can be directed to the corresponding author.
